# Are cardiac R2* values dependent on the image analysis approach employed?

**DOI:** 10.1186/1532-429X-15-S1-P76

**Published:** 2013-01-30

**Authors:** Antonella Meloni, Hugh Y Rienhoff, Amber Jones, Alessia Pepe, Massimo Lombardi, John C Wood

**Affiliations:** 1 CMR Unit, Fondazione G.Monasterio CNR-Regione Toscana and Institute of Clinical Physiology, Pisa, Italy; 2 Department of Pediatrics, Division of Cardiology, Children's Hospital Los Angeles, Los Angeles, CA, USA; 3 FerroKin BioSciences, Inc, San Carlo, CA, USA; 4 Department of Radiology, Children's Hospital Los Angeles, Los Angeles, CA, USA

## Background

CMR R2* is the gold standard for monitoring cardiac iron overload in patients with hemoglobinopathies. The R2* value is obtained by fitting the signal at different echo times (TEs) to an appropriate decay model. Patients with heavy cardiac iron burden (R2*>100 Hz) exhibit rapid signal, leading to a plateau in the later images. Two approaches have been used to address this. The first one (truncation model) consists in discarding the late "plateau" points and fitting the remaining ones with a single exponential model. The second approach is to fit the signal to an exponential decay plus a constant offset (Exp-C).

We aimed to determine whether systematic differences were present between R2* values obtained with these two approaches.

## Methods

Single-center cohorts were used to compare black blood and bright sequences separately and a multi-center cohort of mixed bright and black blood studies was used to compare robustness and generalizability of the comparison. The R2* value within a region of interest (ROI) drawn in mid-ventricular septum was assessed using each of the two methods in turn. Truncated exponential estimates were calculated with CMRTools that uses a region-based approach (R2*CMRTools). Exp-C estimates were calculated using a rapid pseudo-pixelwise (PPW) implementation written in MATLAB. The mean and the median (R2*PPW-mean and R2*PPW-median) from the R2* distribution were obtained. To distinguish whether differences in measured R2* values resulted from the underlying fitting model or from the use of a PPW rather than a region-based approach, we performed Exp-C fits to a single ROI (R2*PPW-ROI_based).

## Results

Table 1 shows the results for the two methods. No differences could be distinguished based upon whether a white or black blood sequence was examined. The two fitting algorithms gave similar R2* values, with R-squared values exceeding 0.997 and CoV of 3-4%. Results using the PPW method yielded a small systematic bias that became apparent in patients with severe iron deposition. This disparity disappeared when Exp+C fitting was used on a single ROI suggesting that the use of pixelwise mapping was responsible for 3% bias. In the multicenter cohort the strong agreement between R2* values obtained with the two approaches was reconfirmed.

**Table 1:** 

	Paired t-test		Regression Analysis				Bland Altman		CoV (%)
		
	Mean Values (Hz)	P	Slope	P for Slope ≠1	Intercept (Hz)	R-squared	Mean diff (Hz)	Limits (Hz)	
***a) First single-center cohort: black blood images (N=42)***									

R2* _Iron-mean_ vs R2* _CMRTools_	48.5±54.7 vs 48.2±53.1	0.945	1.031±0.005	<0.0001	1.176±0.379	0.999	0.3	4.4 to 5.1	3.53
R2*_Iron-median_ vs R2*_CMRTools_	48.9±55.0 vs 48.2±53.1	0.258	1.036±0.007	<0.0001	-1.007±0.473	0.998	0.7	- 5.0 to 6.5	4.37
R2*_Iron-ROI_based_ vs R2*_CMRTools_	48.9±52.7 vs 48.2±53.1	0.063	0.993±0.007	0.336	1.058± 0.512	0.998	0.7	-4.1 to 5.5	3.66

***b) Second single-center cohort: bright blood images (N=70)***									

R2* _Iron-mean_ vs R2* _CMRTools_	47.6±37.9 vs 47.2±36.9	0.088	1.025±0.005	<0.0001	-0.741±0.300	0.998	0.4	-3.1 to 3.9	2.70
R2*_Iron-median_ vs R2*_CMRTools_	47.7±38.2 vs 47.2±36.9	0.085	1.035±0.006	<0.0001	-1.190±0.373	0.998	0.4	-4.0 to 4.9	3.45
R2*_Iron-ROI_based_ vs R2*_CMRTools_	47.5±37.4 vs 47.2±36.9	0.050	1.013±0.004	0.002	-0.245±0.247	0.999	0.4	-2.3 to 3.0	2.06

***c) Multi-center cohort (N=62)***									

R2* _Iron-mean_ vs R2* _CMRTools_	43.5±22.6 vs 43.8±22.7	0.250	0.989±0.008	0.148	0.108±0.372	0.997	-0.4	-3.0 to 2.3	2.25
R2*_Iron-median_ vs R2*_CMRTools_	43.7±22.4 vs 43.8±22.7	0.989	0.982±0.007	0.015	0.705±0.359	0.997	-0.1	-2.7 to 2.6	2.25
R2*_Iron-ROI_based_ vs R2*_CMRTools_	44.0±22.5 vs 43.8±22.7	0.207	0.989±0.007	0.131	0.681±0.352	0.997	0.2	-2.3 to 2.7	2.07

## Conclusions

Cardiac R2* values are independent of the signal model used for its calculation over clinically relevant ranges; pixelwise fitting generate insignificantly greater R2* estimates at high iron concentrations. The overall variability between the techniques is exceeding small allowing clinicians to compare results with confidence.

## Funding

This work was supported by a grant from the National Institutes of Health, National Heart Lung and Blood Institute (1 RO1 HL075592-01A1) and FerroKin BioSciences as sponsor of the study.

